# Comparison of research outputs and clinical competence between orthopedic surgeons from two training pathways: A single-center study at a Chinese tertiary general hospital

**DOI:** 10.1371/journal.pone.0358770

**Published:** 2026-09-25

**Authors:** Zhijian Li, Lan Wang

**Affiliations:** 1 Department of Human Resource, Shanghai Sixth People’s Hospital affiliated to Shanghai Jiao Tong University School of Medicine, Shanghai, China; 2 Hospital Management Research Center, Shanghai Sixth People’s Hospital affiliated to Shanghai Jiao Tong University School of Medicine, Shanghai, China; University of Marburg: Philipps-Universitat Marburg, GERMANY

## Abstract

**Background:**

In China, there are two primary models for medical doctorate training. This study seeks to conduct a comparative analysis of the research outputs and clinical competence for orthopedic doctoral surgeons under these two models, aiming to provide a reference for the further optimization of training programs.

**Methods:**

Basic information, research output, and clinical performance data were systematically collected from orthopedic surgeons employed at the National Center for Orthopedics of a public tertiary hospital from January 2013 to December 2022. Inter-group comparisons were conducted using the Chi-square test and Mann-Whitney U test. Binary logistic regression analysis was additionally adopted to quantify the magnitude of the association between medical training background and research outputs.

**Results:**

The median ages of orthopedic surgeons from the eight-year program and traditional medical education backgrounds were 31.0 years and 34.5 years, respectively (p < 0.001). After adjusting for gender and seniority, orthopedic surgeons with traditional medical education significantly outperformed their eight-year program counterparts in leading national-level research projects (OR=5.13, 95%CI:1.55–16.97) and provincial/ministerial-level research projects (OR=4.68, 95%CI: 1.38–15.92). However, the two groups demonstrated comparable performance in terms of surgical difficulty and volume.

**Conclusion:**

Orthopedic surgeons from the eight-year program exhibit a more accelerated career progression compared to those from traditional medical education backgrounds. However, their research potential requires further development. Efforts should be made to enhance their research literacy and innovation capability while maintaining clinical performance.

## Introduction

Currently, China maintains two parallel pathways for medical doctoral education and training, each with unique and mutually complementary strengths [1]. Both eight-year program and traditional medical education have made significant contributions to the cultivation of medical professionals [2]. Traditional medical education, as one of the mainstream training models for medical doctors, has a long developmental history and has established a relatively mature and comprehensive training system. In response to the need for deepening long-cycle clinical medical education reform and adapting to national medical innovation demands and international competition, eight-year medical education has emerged as an alternative model aimed at cultivating high-level medical professionals with innovative thinking and a global perspective [3]. However, how to efficiently achieve these training objectives and fully realize the potential of students remains a critical challenge requiring further exploration.

Eight-year program medical education refers to a streamlined clinical medicine training model that integrates undergraduate and doctoral studies in a continuous eight-year curriculum. In contrast, obtaining a doctoral degree through traditional medical education in China typically requires 11 years or more [2]. Since China fully implemented eight-year program medical education in 2004, a total of 18 institutions have enrolled students under this model [4]. In September 2020, the State Council issued the Guidelines on Accelerating the Innovative Development of Medical Education, which called for promoting integrated eight-year clinical medicine education reform that bridges basic and clinical disciplines, with the aim of cultivating high-level interdisciplinary talents [5]. Eight-year program medical education constitutes a significant component of China’s medical talent cultivation system. As one of the main channels for cultivating high-level, innovative medical talents in China, the developmental trajectory of eight-year program graduates is closely intertwined with the long-term progress of China’s healthcare system [6]. However, a clear understanding of their current developmental status and research potential remains lacking. Existing studies have primarily focused on curriculum exploration [7], student teaching demands [8], and differences between various training models. Some studies have also analyzed the employment status and career trends of eight-year program medical graduates [9]. However, research investigating the post-graduation career development in hospitals among eight-year program graduates remains relatively scarce.

The tertiary public hospital enrolled in this study holds official accreditation as the National Center for Orthopedics. It has consistently recruited orthopedic medical students from both eight-year programs and traditional medical education tracks, making it representative of the real-world profiles of orthopedic physicians practicing at top-tier medical institutions in China. This study aimed to compare differences in research output and clinical competence among orthopedic surgeons between two medical doctoral training models, summarize their respective strengths and limitations, and provide evidence-based references for physician training optimization and career pathway planning.

## Materials and methods

### Research subjects

The participants of this study were orthopedic surgeons practicing at the National Center for Orthopedics affiliated with a tertiary general hospital in Shanghai. Subjects were selected based on the following inclusion and exclusion criteria. Inclusion criteria: (1) employment start date between 1 January 2013 and 31 December 2022; (2) possession of a doctoral degree; (3) clinical orthopedic surgeons. Exclusion criteria: (1) orthopedic surgeons with only a master's degree or lower academic qualifications. A total of 38 orthopedic surgeons from the eight-year program and 46 from the traditional program were ultimately included in the study. All participants were analysed anonymously.

### Data collection

This study systematically collected information on the basic characteristics, clinical performance, and research output of each orthopedic surgeon. To ensure scientific validity and reliability, all data were uniformly exported from the hospital's information statistics system. (1) Basic characteristics: primarily included indicators related to personal career development, such as age, professional title, and years of work experience. (2) Clinical performance: the indicator was assessed via two dimensions—workload quantity and service quality, referencing the hospital's routine framework for evaluating physicians' clinical performance. Regarding workload quantity, outpatient visits and surgical volume are universally recognized as readily accessible, high-precision metrics for clinical activity. However, outpatient attendance is substantially confounded by fixed scheduling arrangements; therefore, only surgical volume was incorporated into the analysis. For the dimension of clinical quality, the Case Mix Index (CMI) quantifies the complexity of admitted conditions and the overall technical difficulty of medical care delivery. A higher CMI value indicates that a physician manages more complicated and technically demanding orthopedic cases [10]. It is also an official indicator specified in the 2025 Edition of National Standards for Tertiary Hospital Accreditation to assess hospital operational performance. The computational formula for CMI is presented below: $CMI=\frac{\sum ({Number\;of\;cases}_{i}\times DRG\;{relative\;weight}_{i})}{Total\;inpatient\;cases}$, each DRG group is assigned a relative weight corresponding to operative complexity, patient comorbidities and resource consumption. The number of clinical surgical cases and the Case Mix Index (CMI) for each orthopedic surgeon in 2023 were collected. (3) Research output: This was primarily assessed based on two aspects: research projects undertaken and papers published in the Science Citation Index (SCI). This study only included research projects supported by government. All SCI-indexed publications were categorized into four tiers (Q1, Q2, Q3, and Q4) according to the quartile ranking of the Journal Citation Reports (JCR). All journals were categorized based on their respective disciplinary classifications, with no instances of overlapping quartile assignments.

### Statistical analysis

Data were organized using Excel, and statistical analyses were performed using SPSS version 27.0. The significance level was set at 0.05 (two-tailed). Normality of continuous variables was assessed using the Shapiro–Wilk test. Continuous variables conforming to a normal distribution were expressed as mean ± standard deviation, and inter-group comparisons were conducted using independent samples t-tests. Continuous variables with non-normal distribution were presented as median and interquartile range [M (P25, P75)], and inter-group differences were analyzed using the Mann-Whitney U test. Categorical variables were expressed as frequencies and composition ratios [n (%)], with inter-group comparisons performed using the chi-square test. Binary logistic regression analysis was performed to explore the association between medical training background (eight-year program versus traditional medical training pathway) and research output. Multiple models with covariate adjustments were constructed to control for potential confounding factors. Notably, post-employment working duration demonstrated a stronger association with research productivity than age in the present study, since all research productivity data were measured after employment. Accordingly, length of employment was incorporated as the primary confounding variable.

## Results

### Analysis of basic characteristics

No statistically significant differences were observed between orthopedic surgeons trained under the eight-year programme and those with a traditional medical education background regarding the distribution of sex, professional title, or length of employment. Notably, both educational background groups exhibited a high proportion of male physicians, accounting for 94.7% and 97.8%, respectively. Regarding professional title distribution, eight-year program orthopedic surgeons with intermediate and junior titles constituted nearly 50% of the group, whereas among non-eight-year program orthopedic surgeons, the proportion of junior titles (50.0%) exceeded that of intermediate titles (30.1%). Furthermore, the median age of eight-year program and non-eight-year program orthopedic surgeons was 31 years and 34.5 years, respectively, with the difference being statistically significant (p < 0.001), as detailed in Table 1.

**Table 1:** Basic characteristics of eight-year program and non-eight-year program orthopedic surgeons.

Basic Characteristics	Eight-Year Program	Non-Eight-Year Program	x^2^/z-value	P-value
**Sex**			0.028	0.866
Male	36 (94.7)	45 (97.8)		
Female	2 (5.3)	1 (2.2)		
**Professional Title**			2.398	0.303
Junior	18 (47.4)	23 (50.0)		
Intermediate	19 (50.0)	18 (30.1)		
Senior	1 (2.6)	5 (10.9)		
**Age(years)**			−4.873	<0.001
Median	31.0	34.5		
Interquartile Range	(30.8,34.3)	(33.0,36.3)		
**Length of employment**			−0.927	0.354
Median	5.5	6.5		
Interquartile Range	(4.5,7.5)	(4.5,7.8)		

Note: Sex and professional title are presented as composition ratios (n(%)), while age and length of employment are presented as [M(P25, P75)].

### Analysis of clinical performance between eight-year program and non-eight-year program orthopedic surgeons

Physicians with junior titles generally do not qualify as operating surgeons, so we only included surgical procedures performed by physicians with intermediate titles or above in the analysis. The median CMI value for surgical procedures was 1.2 for both eight-year program and non-eight-year program surgeons, with no statistically significant difference observed (p > 0.05). Similarly, no statistically significant differences were found in total surgical volume or the number of Grade III and IV surgical procedures between the two groups. Regarding surgical assistance, the number of procedures in which orthopedic surgeons served as first assistants also showed no statistically significant difference between the eight-year program and non-eight-year program groups (p > 0.05). These findings indicate that orthopedic surgeons from the two educational backgrounds demonstrated comparable clinical level. Detailed results are presented in Table 2.

**Table 2:** Comparative analysis of surgical procedures between eight-year program and non-eight-year program orthopedic surgeons [M(P25, P75)].

Surgical Performance	Eight-Year Program	Non-Eight-Year Program	Z-value	P-value
Lead Surgeon CMI	1.2 (0.9,1.4)	1.2 (1.1,1.4)	−0.828	0.408
Number of Surgeon Procedures	8.0 (3.5,63.0)	29.5 (2.0,98.5)	−0.928	0.354
Number of Grade Ⅲ/IV Surgeon Procedures	1.0 (0,35.0)	10.50 (0,42.8)	−1.273	0.203
Number of First Assistant Procedures	218.0 (54.3,503.3)	165.0 (21.5,353.3)	−0.699	0.484
Number of Grade Ⅲ/IV First Assistant Procedures	107.0 (6.0,257.0)	108.5 (13.5,288.8)	−0.466	0.641

### Comparative analysis of research outputs among orthopedic surgeons with eight-year program and non-eight-year program

Chi-square test results showed that, regarding research projects, non-eight-year program orthopedic surgeons significantly outperformed their eight-year program counterparts both provincial/ministerial-level and national-level projects, with statistically significant differences observed(p < 0.05). Overall, the proportion of research projects non-eight-year program orthopedic surgeons was significantly higher than that of eight-year program orthopedic surgeons received (p < 0.05). In terms of SCI publications, the number of non-eight-year program orthopedic surgeons publishing high-quality papers in Q1 journals was significantly higher than that of eight-year program orthopedic surgeons (p < 0.05). as detailed in Table 3.

**Table 3:** Chi-square analysis of longitudinal research projects and papers: orthopedic surgeons with eight-year vs. non-eight-year medical programs(n(%)).

	Eight-Year Program	Non-Eight-Year Program	χ²-value	P-value
**Projects Level**				
**National-Level**			8.007	0.005
Received	13 (34.2)	30 (65.2)		
Not received	25 (65.8)	16 (34.8)		
**Provincial-Level**			8.808	0.003
Received	4 (10.5)	18 (39.1)		
Not received	34 (89.5)	28 (60.9)		
**Bureau-Level**			0.483	0.487
Received	8 (21.1)	7 (15.2)		
Not received	30 (78.9)	39 (84.8)		
**Any Level**			7.352	0.007
Received	19 (50.0)	36 (78.3)		
Not received	19 (50.0)	10 (21.7)		
**SCI Publications**				
**Q1 Journals**			5.002	0.025
Published	13 (34.2)	27 (58.7)		
Not Published	25 (65.8)	19 (41.3)		
**Q2 Journals**			2.564	0.109
Published	14 (36.8)	25 (54.3)		
Not Published	24 (63.2)	21 (45.7)		
**Q3 Journals**			0.010	0.920
Published	12 (31.6)	15 (32.6)		
Not Published	26 (68.4)	31 (67.4)		
**Q4 Journals**			0.999	0.318
Published	6 (15.8)	4 (8.7)		
Not Published	32 (84.2)	42 (91.3)		
**SCI Papers(Overall)**			3.158	0.076
Published	22 (57.9)	35 (76.1)		
Not Published	16 (42.1)	11 (23.9)		

After covariate adjustment, binary logistic regression analysis revealed that medical educational background (eight-year vs. non-eight-year medical program) was independently correlated with the acquisition of national-level research projects (OR=5.13, 95%CI:1.55–16.97) and provincial/ministerial-level research projects (OR=4.68, 95%CI: 1.38–15.92). For Q1 journal publications, the association with educational background exhibited distinct changes across different adjusted models. Specifically, the association remained statistically significant after adjusting for only the primary confounding variable (P < 0.05), whereas statistical significance vanished following full adjustment for three confounding variables (P > 0.05), as detailed in Table 4.

**Table 4:** Binary logistic regression of research outputs: orthopedic surgeons with eight-year vs. non-eight-year medical programs.

Education model	Received National- Level Projects		Received Provincial- Level Projects		Published in Q1 journals	
	P	OR(95%CI)	P	OR(95%CI)	P	OR(95%CI)
Model 1						
Education background	0.005	3.61 (1.46,8.91)	0.005	5.46 (1.66,18.02)	0.027	2.73 (1.12,6.66)
Model 2						
Education background	0.009	4.10 (1.42,11.8)	0.007	5.29 (1.59,17.56)	0.041	2.70 (1.04,7.02)
Seniority	<0.001	1.76 (1.31,2.36)	0.755	1.04 (0.82,1.32)	0.003	1.46 (1.14,1.87)
Model 3						
Education background	0.007	5.13 (1.55,16.97)	0.013	4.68 (1.38,15.92)	0.059	2.60 (0.97,7.01)
Seniority	0.111	1.43 (0.92,2.20)	0.419	0.85 (0.58,1.26)	0.551	1.12 (0.78,1.59)
Sex	0.999	8.73 × 10^8^ (0.00,∞)	0.999	3.73 × 10^8^ (0.00,∞)	0.981	0.97 (0.08,12.56)
Junior	Ref		Ref		Ref	
Intermediate	0.030	5.61 (1.18,26.76)	0.575	1.57 (0.33,7.53)	0.048	4.07 (1.02,16.33)
Senior	0.921	0.87 (0.05,14.62)	0.099	10.81 (0.64,183.46)	0.180	7.23 (0.40,130.17)

## Discussion

The results of this study indicate that, in terms of years of service and promotion to professional titles, orthopedic surgeons who completed the eight-year programme show no difference compared to those who did not, but their average age is significantly lower (p < 0.05). This indicates that orthopedic surgeons with eight-year programme reach identical professional titles at an earlier career stage. Existing research findings also indicate that the majority of students choose the eight-year programme because it enables them to obtain a PhD more rapidly [11]. this structural training advantage also helps cultivate the high-quality surgeons of the eight-year programme. In addition, candidates admitted to eight-year medical programs typically achieve top-tier college entrance examination rankings, previous studies have confirmed that academically outstanding students generally possess stronger self-awareness of professional orientation and career planning, which facilitates proactive and long-term career development [12]. Combined with the systematic and early academic training embedded in the eight-year curriculum, these inherent student and curriculum advantages jointly accelerate the early career progression of eight-year-trained orthopedic surgeons, without compromising mid-term professional promotion efficiency.

Research innovation serves as a crucial pathway for advancing medical science and technology and facilitating the translation of medical achievement [13]. Research outcomes, such as papers and research projects, are widely recognized as key indicators of research competency. This study found that orthopedic surgeons with eight-year program exhibited superior performance in undertaking national and provincial/ministerial-level research projects compared with conventionally trained physician, while no significant difference was observed in SCI paper publications(P > 0.05). This inconsistent outcome may be explained by the distinct characteristics of different academic achievements, compared with manuscript publication, research project application demands more long-term research accumulation and in-depth academic critical thinking.

Overall, these findings indicate that the research literacy of eight-year-trained orthopedic surgeons remains to be further improved, which is consistent with the conclusions of previous studies [14]. Several factors may account for this disparity. Firstly, orthopedic surgeons should devote most of their energy to clinical practice, which reduces their internal motivation for research and innovation. Secondly, structured research training modules within the eight-year medical pathway require further optimization [15]. This is largely because the eight-year medical education system in China confers a professional doctorate (M.D.), allowing limited time for dedicated research. Given the need to obtain a doctoral degree within a relatively short period, students experience considerable academic pressure [16], further reducing the time available for research.

Furthermore, universities adopt varying training curricula and objectives for research innovation in eight-year medical education at different developmental stages, with some institutions even lacking mandatory publication requirements. This inconsistency impedes the establishment of a cohesive research capacity training model. For instance, since establishing its eight-year medical education program, Peking University has revised its training curriculum five times, with each iteration reflecting shifts in educational philosophy. To enhance research literacy, some experts have proposed that eight-year program medical graduates should undergo an additional 2–3 years of research training and be eligible for a Doctor of Science (Ph.D.) degree in addition to their medical degree [3]. Alternatively, the appeal of research can be further enhanced by enriching the curriculum and introducing research-oriented courses at an earlier stage [17]. In the professional phase, incentive mechanisms should be progressively enhanced to promote research capacity development and facilitate the translation of research findings into clinical practice.

A study has indicated that eight-year program physicians demonstrate lower clinical skills and patient care capabilities compared to their non-eight-year program counterparts during the initial stages of practice [18]. However, the findings of this study reveal no substantial differences in surgical volume or surgical complexity between orthopedic surgeons from the two educational backgrounds, suggesting that the clinical skill level of eight-year program physicians is generally satisfactory after practical training, a conclusion consistent with existing research [19].

The reasons for this are, on one hand, that during the educational training phase, major universities place significant emphasis on clinical curriculum design and clinical skills training. For example, a university has effectively integrated clinical skills training into both its four-year core medical education and its international joint training programs [20], another university, under its“3+2+3”model, dedicates three years to clinical knowledge acquisition in general hospitals [21]. On the other hand, since the nationwide implementation of the standardized residency training system in 2015, eight-year program orthopedic surgeons are still required to complete 2–3 years of clinical residency after graduation [22]. Rotational training across various departments significantly enhances clinical proficiency and helps standardize clinical competencies across different educational backgrounds. Beyond this, some hospitals are exploring new models that integrate clinical competency development for eight-year programme graduates with standardized training of physicians [23].

The findings regarding the career development, clinical competence, and research outputs of orthopedic surgeons with eight-year program provide valuable references for medical educators and policy makers. Nevertheless, several limitations of this study should be acknowledged. First, this is a single-center study, and the results may not be generalizable to other medical specialties or regional medical institutions. Second, the sample size is relatively small, with a particularly limited number of female orthopedic surgeons, which may obscure potential gender-based differences; this phenomenon is consistent with the inherent gender distribution characteristics of orthopedics. Third, the evaluation indicators adopted in this study are primarily derived from routine clinical practice, which inevitably has certain limitations. Furthermore, the indicators for evaluating research output can be further optimized and refined in future studies. Specifically, subsequent research could quantitatively explore the differences in the impact factor of SCI publications among orthopedic surgeons with different educational backgrounds.

## Conclusions

In summary, orthopedic surgeons trained under the eight-year programme are able to master clinical skills in a shorter timeframe and achieve the same career progression as their counterparts with a traditional medical education; however, their capacity for scientific research and innovation remains to be strengthened. As one of the primary channels for cultivating high-calibre medical talent in China, the eight-year medical education model requires further refinement and enhancement, and its value for medical students' career development warrants more in-depth exploration.

## Supporting information

S1 File Dataset. (TXT)
